# Determining the Optimal Length and Safety of Pedicle Screws in the T12 Vertebra: A Morphometric Study

**DOI:** 10.7759/cureus.2156

**Published:** 2018-02-05

**Authors:** Mehmet F Korkmaz, Mehmet N Erdem, Huseyin Ozevren, Reşit Sevimli

**Affiliations:** 1 Department of Orthopaedics and Traumatology, Inonu University School of Medicine, Malatya; 2 Orthopaedics and Traumatology, Hisar Intercontinental Hospital; 3 Department of Neurosurgery, Faculty of Medicine, Dicle University

**Keywords:** computed tomography, pedicle screw length, preoperative planning, t12 vertebra

## Abstract

Introduction: Despite the developments in implant technology and imaging methods and the advances in surgical techniques, there are still potential problems and complications of transpedicular screw application. This is a morphometric study to examine the proximity of the T12 vertebra to the thoracic aorta. Our aim was to define the appropriate length of the pedicle screw to be used in the 12^th^ thoracic vertebra, using computed tomography (CT) data.

Methods: Randomly selected cases from the same ethnic group in a specific age group were examined in terms of the length from the anterior vertebral body and the screw entry point of the T12 vertebra to the thoracic aorta. In light of these data, a statistical analysis was made for the selection of the most appropriate screw length.

Results: A statistically significant difference was detected in the distance from the T12 left screw entry point to the aorta between males and females (p=0.001). No statistically significant correlation was found between age and the distance between the left screw entry point and the aorta (p=0.105). Also, no statistically significant difference was detected between the T12 vertebral body-aorta distance in males and in females (p=0.212). The relationship between the shortest aorta-vertebral body distance and age was not statistically significant (p=0.7). Similarly, there was no statistically significant difference between the left screw entry point-aorta distance and the aorta-vertebral body shortest distance (p=0.731).

Conclusions: Significant differences were observed between males and females in terms of the distance between the T12 vertebra left screw entry point and the thoracic aorta (p=0.001). Thus, we can assert the need for the preoperative evaluation of patients with computed tomography in selecting the appropriate screw length and avoiding complications.

## Introduction

The spinal fixation method based on transpedicular screws was first described by Roy-Camille et al. [1]. Since 1963, the method has become more popular and a basic application of spinal fixation in recent years [2]. Despite the developments in implant technology and imaging methods over this time period and advances in surgical techniques, there are still potential problems and complications of transpedicular screw application [3]. Therefore, improvements in spinal fixation and healthy functional development of the pedicle screw system are important areas to be studied.

The thoracolumbar region is the area of transfer of biomechanical movement in the spine, therefore, it is the area where pathologies are seen most often. These pathologies include trauma, degenerative diseases, osteoporotic compression fractures, instabilities, neoplastic diseases, and infections [4]. The instrumentation of the thoracolumbar region with transpedicular screws is the most current and widespread choice in the treatment of these diseases.

The size of the screw should be determined by the compatibility of the entry point with the anatomical guides, such as the proximity of the thoracic aorta to the vertebral body and particularly at the level of the T12 vertebra. This is extremely important in preventing mortal complications, such as aorta injury.

Our aim in this study was to determine the optimum screw length by examining the T12 vertebra pedicle morphology with computed tomography (CT) and, thereby, to preoperatively calculate its depth.

## Materials and methods

The study was designed as a prospective, randomized, and morphometric study to examine the proximity of the T12 vertebra to the thoracic aorta using a CT scan. The CT sections of the T12 vertebrae of patients aged between 25 and 40 years who presented at the Emergency Department or clinic, for whom a thoracic CT was requested for any possible pathology, were examined. The slices were obtained from only an Anatolian population. Patients with spinal deformity, tumor, fracture, infection, congenital anomaly, or history of surgery in the lumbar region were excluded. The study was completed with 30 males and 30 females who met the inclusion criteria and had a mean age of 31.9±3.9 (range: 25 to 40) years.

First, axial images at 3-mm thickness were obtained. Following reconstruction in the bone window, the measurements of three images where the pedicle thickness was the greatest were taken, and the thickest measurement of the pedicle was selected from these images. Then, the distance between the screw entry point and the thoracic aorta and the distance between the anterior vertebral body and the thoracic aorta were digitally measured on this image with a magnetic resonance imaging (MRI) scanner (Leonardo Workstation Siemens, Siemens AG, Munich, Germany). Axial slices of 3-mm thickness from the thoracic vertebra were taken using a Philips MX 8000 CT unit (Philips Healthcare, Best, Netherlands) and examined.

**Figure 1:**
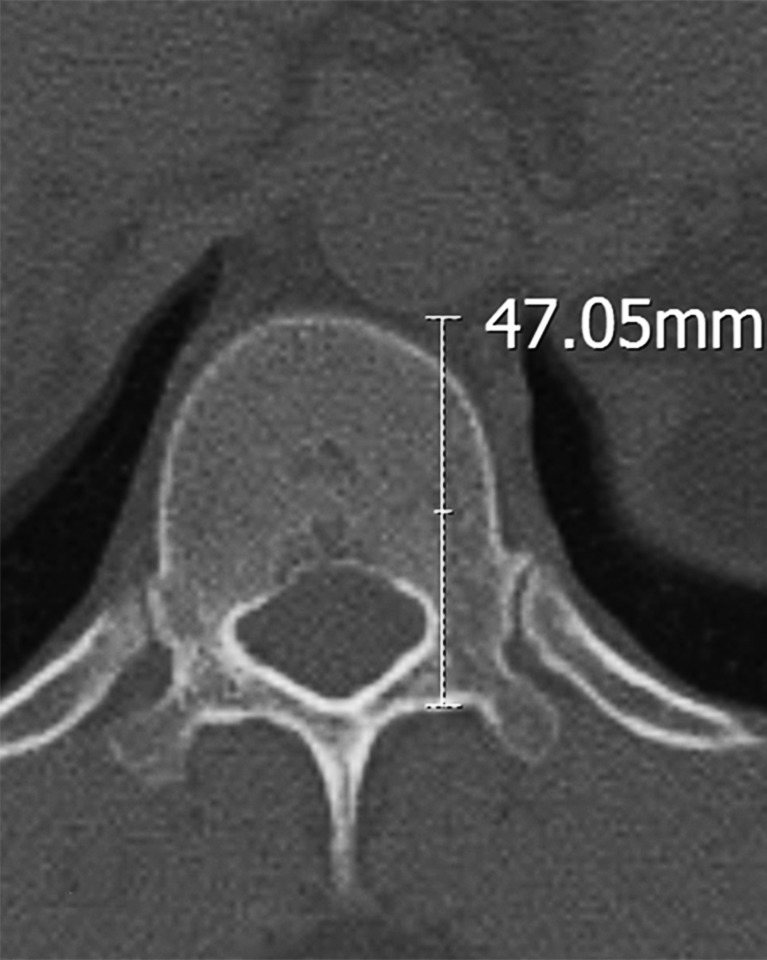
T12 pedicle axial transverse section in a male. The distance between the screw entry point and the thoracic aorta.

**Figure 2:**
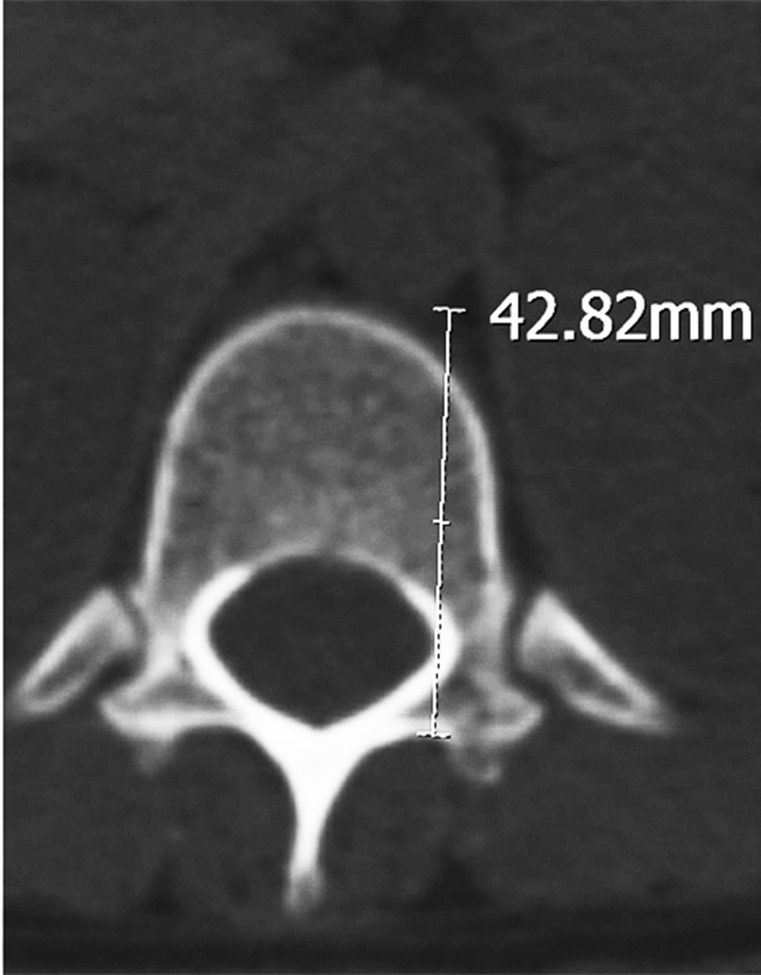
T12 pedicle axial transverse section in a female. The distance between the screw entry point and the thoracic aorta.

Tomography images of the T12 vertebra were taken from the pedicle isthmus at 3-mm thickness (mid-pedicle slice) in the axial plane. The following parameters were examined on the CT slices: the distance between the screw entry point and the thoracic aorta and the distance between the anterior vertebral body and the thoracic aorta. For the length between the left screw entry point and the aorta, the measurement of the closest point of the T12 left screw entry point to the thoracic aorta was made (Figure 1 and Figure 2).

**Figure 3:**
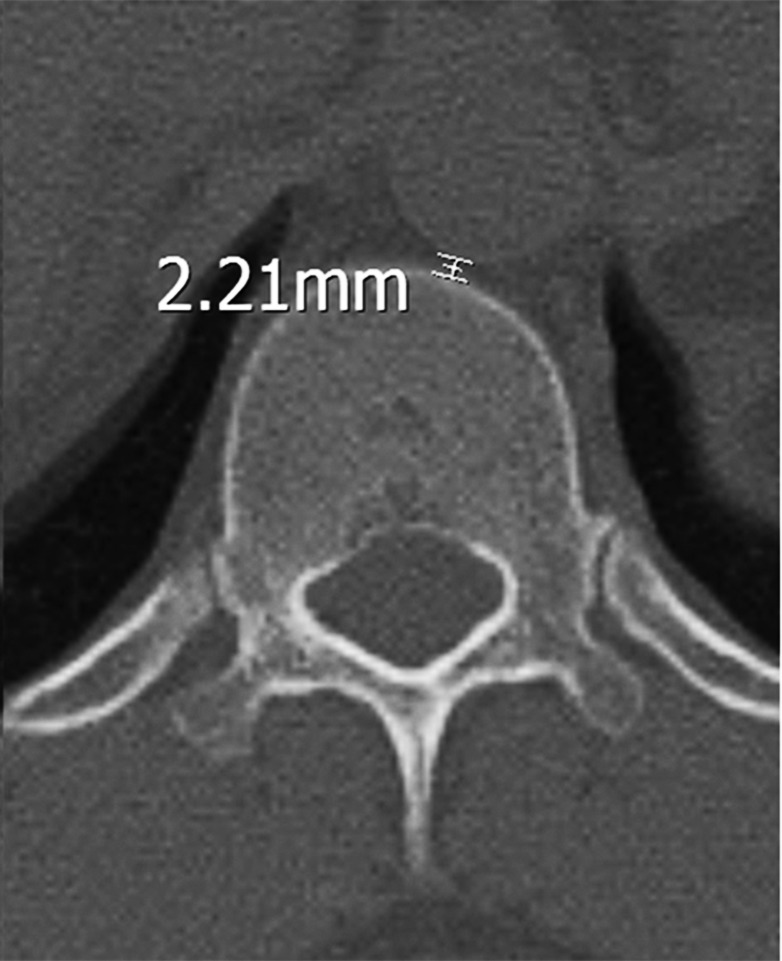
T12 pedicle axial transverse section in a male. The distance between the anterior vertebral body and the thoracic aorta.

**Figure 4:**
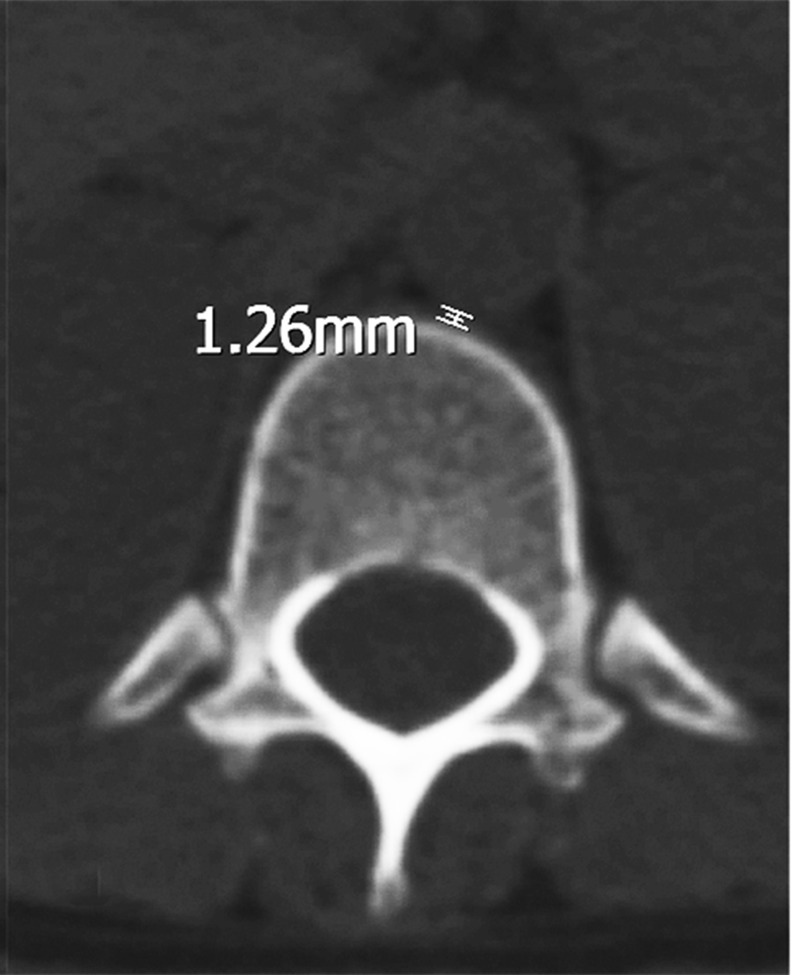
T12 pedicle axial transverse section in a female. The distance between the anterior vertebral body and the thoracic aorta.

For the closest distance between the T12 vertebral body and the aorta, the measurement of the closest distance between the anterior of the T12 vertebral body and the thoracic aorta was made (Figure 3 and Figure 4). 

All data were input into Microsoft Excel and analyses were made using the SPSS software (IBM, Armonk, New York, United States). The measurement results were expressed as mean±standard deviation (SD) and range of values. Statistical analyses were performed with the student’s t-test and Pearson’s correlation coefficient and using the PASW Statistics v.18 software (IBM, Armonk, New York, United States).

## Results

A statistically significant difference was detected in the distance from the T12 left screw entry point to the aorta between males and females. The distance in the males was 47.12±3.38 (range: 40.01 to 54.00) mm and in the females, it was 43.70±3.00 (range: 37.99 to 49.26) mm (p=0.001) (Table 1).

**Table 1:** Comparison of the data of the distance between the left screw entry point and the aorta according to gender.

	Gender	N	Mean	SD	Min-Max	p
Distance between the left screw entry point-aorta (mm)	Male	30	47.12	3.38	37.99- 54.00	0.001
	Female	30	43.70	3.00		

No statistically significant correlation was found between age (31.93±3.91 (range: 25 to 40) years) and the distance between the left screw entry point and the aorta (45.41±3.61 (range: 37.99 to 54.00) mm) (p=0.105) (Table 2).

**Table 2:** Comparison of the data of age and the shortest distance between the left screw entry point and the aorta.

	N	Mean	SD	Min-Max	p
Age	60	31.93	3.91	25-40	0.105
Left screw entry point-aorta shortest distance (mm)	60	45.41	3.61	37.99-54.0	

Also, no statistically significant difference was detected between the T12 vertebral body-aorta distance in males (1.77±0.55 (range: 0.78 to 3.16) mm) and in females (1.94±0.52 (range: 1.02 to 3.56) mm) (p=0.212) (Table 3).

**Table 3:** Comparison of the data of the shortest distance between the T12 corpus and the aorta according to gender.

	Gender	N	Mean	SD	Min-Max	p
Aorta–corpus shortest distance (mm)	Male	30	1.77	0.55	0.78- 3.56	0.212
	Female	30	1.94	0.52		

The relationship between the shortest aorta-vertebral body distance (1.85±0.54 (range: 0.78 to 3.56) mm) and age (31.93±3.91 years (range: 25 to 40) years) was not statistically significant (p=0.7) (Table 4).

**Table 4:** Comparison of the data of age and the shortest distance between the T12 corpus and the aorta.

	N	Mean	SD	Min-Max	p
Age	60	31.93	3.91	25-40	0.70
T12 corpus-aorta shortest distance (mm)	60	1.85	0.54	0.78-3.56	

Similarly, there was no statistically significant difference between the left screw entry point-aorta distance (45.41±3.61 (range: 37.99 to 54.00) mm) and the aorta-vertebral body shortest distance (1.85±0.54 (range: 0.78 to 3.56) mm) (p=0.731) (Table 5).

**Table 5:** Comparison of the data of the shortest distance between the T12 corpus and the aorta and the shortest distance between the left screw entry point and the aorta.

	N	Mean	SD	Min-Max	p
Aorta-corpus shortest length	60	1.85	0.54	0.78-3.56	0.731
Left screw entry point-aorta shortest distance (mm)	60	45.41	3.61	37.99-54.0	

## Discussion

Pedicle screw stabilization in the thoracic region is a widely used method applied in the treatment of several pathologies, such as degenerative diseases, deformities, tumor, and traumatic instability. Knowledge of the vertebral body and pedicle morphology provides great advantages to the surgeon in the application of the pedicle screw during surgery.

During spinal instrumentation, the surgeon must decide on the screw length and its path to obtain the strongest fixation without damaging the neurological and vascular structures adjacent to the pedicle and vertebral body. The ideal techniques for screw advancement are obvious, however, the surgeon generally makes the decision of appropriate thickness and length of screw based on previous experience. When compared to the lumbar vertebra, the placement of the thoracic pedicle screw is more difficult due to its small size, varying entry points, and proximity to neurovascular structures and the spinal cord [5]. Any kind of major deviation causing the perforation of the pedicle or vertebral cortex may lead to neurological and vascular damage. In addition, the entry depth of the screw in the axial plane must be checked. Rates of intraoperative pedicle and/or vertebral body perforations during pedicle screw placement have been reported in the literature, ranging from 5.5% to 39.9% [6-7].

The ideal pedicle screw should have the maximum diameter and length that will not impinge on the pedicle cortical layer and the vertebral body [8]. Recently, there has been an increase in studies regarding the correct placement of screws in spinal surgery [9-13]. In a meta-analysis of 130 studies by Kosmopoulos and Schizas, the authors found out that out of 37,337 pedicle screws, a mean of 8.7% was incorrectly placed [14]. The risk of incorrect placement can be reduced with excellent anatomic knowledge, attention paid to all anatomical marks, careful evaluation of preoperative imaging, and the use of modern equipment during surgery. Attention should be paid to the azygos vein, the intercostal arteries, the vena cava inferior, and the aorta, all of which are at risk during the placement of pedicle screws in the thoracic region. Vanichkachhorn et al. reported cases of major vascular injury, which developed following the removal of incorrectly placed screws in the thoracolumbar region [15]. This case inspired a comprehensive evaluation before the removal of incorrectly oriented screws. Lopera et al. presented a series of six cases with seven artery injuries related to incorrectly applied screw fixation systems in the thoracic region [16].

Computed tomography is the easiest method that gives the most accurate results in the evaluation of vertebral morphometry. Therefore, the majority of studies examining screw pedicle compatibility have used CT scanning, as in the current study [17-19]. The measurements made in this study to determine the appropriate screw length for the T12 vertebra are important with respect to preventing major vascular and vital organ injuries related to anterior cortex perforation. Foxx et al. found that 33 of 680 screws placed in the thoracolumbar area were in contact with major blood vessels, and no symptoms or sequelae associated with vascular contact were observed [20]. However, other authors have emphasized that where a screw is in contact with a blood vessel and continually striking it, a secondary lesion (lacerations or pseudoaneurysms) may develop in the area of contact, and a revision surgery will be necessary [21]. Therefore, preoperative determination of appropriate screw length prevents the risk of anterior cortex perforation and major vascular injuries.

This study shows the importance of the length and safety of T12 pedicle screws, evaluates the average values of pedicle screw length, and increases awareness of it. But, the length of the pedicle screws should be measured in the patient’s CT images or plain X-ray before surgery and determined based on the morphology of each patient. This is the major limitation of our study.

## Conclusions

In conclusion, regardless of the technique applied, pedicle-screw-based instrumentation is a strong fixation method for the thoracolumbar spine. When placing the pedicle screw in the T12 vertebra, the length of the screw is just as important as the screw entry point and orientation. Therefore, preoperative radiological planning and distance measurement will reduce iatrogenic vascular injuries.
